# A review of sarcoidosis etiology, diagnosis and treatment

**DOI:** 10.3389/fmed.2025.1558049

**Published:** 2025-02-26

**Authors:** Yahya Mostafa Waly, Abu-Baker Khalid Sharafeldin, Muhammad Umair Akhtar, Zaid Chilmeran, Salim Fredericks

**Affiliations:** School of Medicine, Royal College of Surgeons in Ireland, Busaiteen, Bahrain

**Keywords:** sarcoidosis, granulomas, diagnosis, etiology, personalized treatment

## Abstract

Sarcoidosis is an inflammatory disease characterized by the formation of granulomas in various organs, leading to inflammation and potential organ dysfunction. Symptoms often start with general signs like fatigue, fever, and weight loss, but vary depending on the affected organ. Diagnosis is challenging due to its diverse clinical presentation and lack of a definitive test, while treatment is complicated by the disease’s variable course, requiring a personalized approach. This review explores the role of genetic and environmental factors in sarcoidosis etiology, examines current challenges in diagnosis and treatment, and discusses how understanding etiology informs patient management and future treatment strategies.

## 1 Introduction

Sarcoidosis is an inflammatory disease marked by the development of small clusters of immune cells known as granulomas, which can form in various organs and tissues throughout the body. These granulomas cause inflammation and may impair the function of the affected organs. While the lungs and lymph nodes are most commonly affected, sarcoidosis can also involve the eyes, skin, heart, and other organs (1).

The disease typically presents with general symptoms such as fatigue, fever, and unintentional weight loss. Additional symptoms depend on the specific organs affected, reflecting the diversity of its clinical manifestations (2).

The challenges associated with diagnosing and treating sarcoidosis have led to an increase in prevalence since 1990 (3). One of the primary difficulties in diagnosing sarcoidosis is due to the lack of a specific test, as no single test can definitively confirm sarcoidosis (4). Moreover, treatment can be challenging due to the variable course of the disease, necessitating a personalized approach (5).

This review explores the role of genetic and environmental factors in the etiology of sarcoidosis, examines current challenges in diagnosis and treatment, and discusses how understanding etiology informs patient management and future treatment strategies.

## 2 Etiology

### 2.1 Genetic factors

Recent studies have illustrated that Human Leukocyte Antigen (HLA) gene associations play a critical role in the pathogenesis of sarcoidosis (6). HLA class II alleles entail variable associations with sarcoidosis depending on geographic and ethnic variability. HLA-DRB1*01 and DRB1*04 were found to have protective effects against sarcoidosis in Caucasian populations, whilst HLA-DRB1*03, DRB1*11, DRB1*12, DRB1*14, and DRB1*15 were identified as risk factors for the disease (7, 8). Löfgren’s syndrome, an acute sarcoidosis phenotype, was proven to be associated with the HLA-B8/DR3 haplotypes (9). Furthermore, studies found that amongst African Americans, the HLA-DQB1 alleles seemed to have a more significant role in sarcoidosis susceptibility than the HLA-DRB1 alleles (10).

Another genetic component that is associated with sarcoidosis is familial predisposition. It has been shown that there is a 3.7 times higher risk of developing the disease for first-degree relatives of patients with sarcoidosis (11). Additionally, studies conducted on twins illustrate a higher concordance rate in monozygotic twins compared to dizygotic twins, suggesting a genetic influence (12).

### 2.2 Occupational and environmental triggers

Some occupational and environmental exposures are associated with an increased risk of developing sarcoidosis. These exposures can be classified into inorganic particles (e.g., silica and beryllium) and airborne exposures (e.g., wood smoke and organic dust).

Miners and construction workers exposed to silica are more likely to develop sarcoidosis-like granulomatous disease (13). Exposure to beryllium in an industrial setting can lead to chronic beryllium disease, which mimics and shares features of sarcoidosis in genetically predisposed individuals (13). Another study confirmed that farmers and individuals exposed to wood-burning stoves or organic dusts have demonstrated a higher prevalence of sarcoidosis (14). Similarly, a study examining the incidence and progression of sarcoidosis among firefighters who responded to the 9/11 attacks found that 74 firefighters developed the disease post-9/11, with an incidence of 25 per 100,000 (15).

### 2.3 Infection

Infection is considered one of the potential factors contributing to the development of sarcoidosis. For example, infectious agents such as mycobacteria and *Propionibacterium* species are associated with sarcoidosis since the formation of granulomas is a necessary component of the immune defense to combat them (16).

Some studies have identified mycobacterial DNA/antigens within the sarcoid granulomas, suggesting a possible correlation in the pathogenesis of the disease. This was further proven by detecting genes such as IS6110 (a marker of *M. tuberculosis*) in granulomatous samples from sarcoidosis patients. In addition, the mycolic acid within the cell wall of the Mycobacterium tuberculosis complex has been proposed as a potential trigger that can elicit an immune response. More specifically, the initiation of a T-helper type 1 (Th1) immune response, which is central to granuloma formation in sarcoidosis, suggesting a similar pathogenesis (17).

Furthermore, studies have found *Cutibacterium* acnes DNA in sarcoid granulomas, especially in tissues like the lungs and lymph nodes; this is thought to drive chronic inflammation and granuloma formation in sarcoidosis (18). Other microorganisms have been identified to have a potential trigger of the immune response in sarcoidosis such as *Borrelia burgdorferi*, herpes virus, retrovirus, *Leptospira* species, and *Chlamydia pneumoniae* (19). However, *Mycobacterium* has been identified as the leading candidate in infection-related sarcoidosis (20).

### 2.4 Immunopathogenesis

**FIGURE 1:**
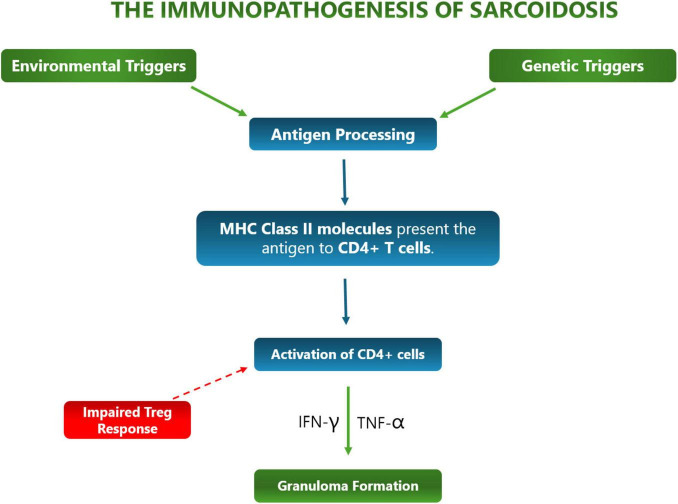
The immunopathogensis of sarcoidosis.

The immunopathogenesis of sarcoidosis (Figure 1) is characterized by a dysregulated response to the many causative antigens contributing to it, leading to granuloma formation and chronic inflammation.

Immunopathogenesis is initiated by antigen presentation, when antigen-presenting cells (APCs) such as macrophages and dendritic cells present antigens to CD4+ T-cells, leading to granuloma formation – the hallmark of sarcoidosis. Furthermore, these APCs trigger an exaggerated Th1 immune response, which produces high levels of Tumor Necrosis Factor alpha (TNF-α) and secrete interleukins 12, 15, and 18, leading to persistent granuloma and chronic inflammation (21). This can be fatal as persistent granulomas and chronic inflammation from sarcoidosis are associated with tissue damage and impaired organ function (22).

## 3 Diagnosis

### 3.1 Conventional diagnostic approaches

Diagnosing sarcoidosis remains a complex and often protracted process due to the need for invasive procedures and the absence of a definitive diagnostic test. It typically requires a combination of clinical, radiological, and histopathological assessments to arrive at a probable diagnosis.

The process of diagnosing sarcoidosis is complicated by the lack of standardization and hinges on three primary criteria: a clinical presentation consistent with sarcoidosis, histological evidence of granulomatous inflammation in at least one tissue, and the exclusion of alternative causes of granulomatous disease (5). Importantly, the diagnosis is based on probability rather than certainty. It is made when the likelihood of other conditions with similar symptoms becomes sufficiently low (23).

Radiological imaging plays a crucial role in the diagnostic workup. Chest X-rays are abnormal in the majority of patients with sarcoidosis and frequently reveal bilateral hilar lymphadenopathy (24, 25). The extent of hilar lymph node enlargement varies, but a symmetric pattern is particularly indicative of sarcoidosis, as it distinguishes the disease from other differential diagnoses such as lymphoma, tuberculosis, and metastatic malignancies. Chest X-ray findings are often classified using the Scadding stages, which range from Stage I (only nodal enlargement) to Stage IV (pulmonary fibrosis). Stage I is the most prevalent, found in approximately 45–65% of patients (25). Computed tomography (CT) offers superior sensitivity in detecting parenchymal abnormalities and calcification patterns in lymph nodes, such as eggshell or sugar-like calcifications. CT imaging also facilitates procedures like transbronchial biopsies by improving localization and diagnostic yield (25). Additionally, FDG-PET and PET/CT have potential value as a useful tool in targeting treatment, as they can assess treatment response and guide therapeutic decisions in patients with sarcoidosis. For example, studies have shown that FDG-PET/CT can monitor the effectiveness of corticosteroids and biologic treatments like infliximab and adalimumab, identifying patients who are responding to therapy and those who may require alternative treatments (26).

A key aspect of the diagnostic pathway involves obtaining tissue samples through procedures such as bronchoalveolar lavage and biopsy to confirm the presence of non-necrotizing granulomas (27). In such cases, advances in minimally invasive techniques, such as endobronchial ultrasound-guided transbronchial needle aspiration, have enhanced the diagnostic accuracy for mediastinal and hilar lymphadenopathy (28).

### 3.2 Role of biomarkers

Given the complexities involved in the diagnosis of sarcoidosis, the identification of reliable biomarkers has become an area of intense research. Current biomarkers are not specific for sarcoidosis and traditionally used biomarkers are not without limitations (29). Serum angiotensin-converting enzyme (ACE) and soluble interleukin-2 receptor (sIL-2R) are the traditional biomarkers routinely assessed in clinical practice (30). ACE is commonly elevated in sarcoidosis, reflecting granulomatous inflammation, but its lack of specificity limits its utility, as it can also be elevated in other conditions like tuberculosis and tumors (31). Likewise, sIL-2R is a marker of T-cell activation, which has been associated with disease activity, but broad overlaps can exist in sIL-2R levels in interstitial lung diseases (32).

In addition to these traditional biomarkers, there is growing interest in the identification of more specific biomarkers that can provide greater diagnostic clarity. Recent studies have highlighted the potential of biomarkers such as specific cytokines, chemokines, and proteins in blood or bronchoalveolar lavage fluid (33). Elevated levels of IL-2, TNF-α, and other pro-inflammatory molecules are frequently observed in sarcoidosis and may serve as indicators of disease activity or progression (34). Emerging pathways such as JAK/STAT signaling have garnered attention, as they play a crucial role in granuloma formation and are differentially expressed in sarcoidosis patients. The identification of specific activation markers within the JAK/STAT pathway, such as those regulated by STAT1 and STAT3, could eventually serve as biomarkers to guide treatment with JAK/STAT inhibitors like tofacitinib or ruxolitinib, which have shown success in therapy-refractory cases (27). Similarly, the mechanistic target of rapamycin (mTOR) signaling pathway, implicated in granuloma formation and progressive disease, presents another area of interest. Markers of mTORC1 activation may serve as therapeutic biomarkers, potentially predicting response to treatments like rapamycin (27), which has displayed success in sarcoidosis treatment (35). In addition to molecular pathways, innovative approaches such as hair cortisol analysis are emerging as non-invasive, long-term biomarkers. Measuring cortisol levels in hair samples can retrospectively assess chronic stress and psychological distress in sarcoidosis patients, providing insights into fatigue and quality of life (27). Despite the promise of these markers, none have yet achieved the ideal sensitivity and specificity needed for widespread clinical adoption (25) (Table 1).

**TABLE 1:** Summary of the role of some biomarkers in sarcoidosis diagnosis and management.

Biomarker	Description	Limitations/Challenges
Angiotensin-converting enzyme (ACE)	Elevated in sarcoidosis, reflects granulomatous inflammation.	Lacks specificity, also elevated in conditions like tuberculosis and tumors.
Soluble interleukin-2 receptor (sIL-2R)	Marker of T-cell activation, linked to disease activity.	Broad overlap in levels with other interstitial lung diseases.
IL-2, TNF-α, pro-inflammatory molecules	Elevated in sarcoidosis, indicating disease activity or progression.	Not specific to sarcoidosis alone.
JAK/STAT signaling	Involved in granuloma formation, STAT1/STAT3 may serve as biomarkers for JAK/STAT inhibitors.	Requires further research and validation for routine clinical use.
mTOR signaling	Implicated in granuloma formation and disease progression, mTORC1 activation may predict response to rapamycin.	
Hair cortisol analysis	Non-invasive biomarker for chronic stress and psychological distress.	

### 3.3 Emerging tools

In recent years, novel diagnostic tools have been developed to improve the accuracy and precision of sarcoidosis diagnosis. Advances in omics technologies, including transcriptomics, radiomics, and pharmacogenetics, have provided new insights into the mechanisms of sarcoidosis (36). For example, transcriptomic studies have focused on gene expression profiling in peripheral blood, bronchoalveolar lavage, and lung tissue, revealing key roles of the TH1 immune response and IFN-g-driven pathways. Additionally, radiomics, which involves the quantitative analysis of HRCT and FDG-PET scans, has shown promise in differentiating sarcoidosis from other conditions while correlating with pulmonary function (37). Furthermore, emerging areas like pharmacogenetics and radiotranscriptomics, which combine genetic analysis with radiological data, may offer new approaches for individualized treatment and disease management. These tools are particularly useful when biopsy is not feasible or in cases of isolated organ involvement, such as in cardiac or neurological sarcoidosis (36).

## 4 Treatment

### 4.1 Conventional therapies

In those who are affected chronically by the disease, corticosteroids are the current first line treatment. For instance, guidelines recommend 20–40 mg per day of oral prednisolone for pulmonary sarcoidosis, which is also the most common manifestation of sarcoidosis (38, 39). This usually continues for 1–3 months followed by tapering to around 10 mg/day as was investigated in a Delphi consensus study (40). Furthermore, one study found there to be no significant difference in the treatment failure or relapse rates as well as adverse events from the medication between groups on higher doses of prednisolone compared to lower doses (41).

Corticosteroids bind intracellular glucocorticoid receptors and induce a conformational change, allowing the complex to enter via nuclear pores (42). Once inside the nucleus, the complex is able to bind to target DNA sequences (glucocorticoid response elements) to stimulate or inhibit gene expression of certain proteins (42). Corticosteroids downregulate the expression of inflammatory genes and pathways such as nuclear factor kappa-B and activator protein-1, leading to a reduction in inflammatory cytokines TNF-α and interleukins 1 and 6 (43). They also inhibit the arachidonic acid pathway by upregulating the lipocortin-1 gene, an anti-inflammatory protein, decreasing production of pro-inflammatory proteins. The anti-inflammatory ability could lead to the suppression of granulomas formed in sarcoidosis. Another important mechanism carried out by corticosteroids is the inhibition of fibroblast activity, leading to reduced collagen deposition and reduced fibrosis which is a complication that can arise in chronic long-standing pulmonary sarcoidosis (44).

Certain patients who suffer a more severe disease compared to others, would require higher dosages of the corticosteroid. This coupled with the chronicity of sarcoidosis requiring long term management of the corticosteroid, can lead to many adverse events arising from the drug. A systematic review detailing common adverse effects of long term corticosteroid use included nausea, cataracts, vomiting, cardiac conditions, hyperglycemia (type 2 Diabetes), and more commonly osteoporosis and hypertension (45). As a result, it may be recommended in these patients to be given corticosteroid sparing drugs, such as methotrexate, rather than corticosteroids to steer clear from the aforementioned side effects (38).

Patients initiating corticosteroid therapy should undergo screening and, if necessary, treatment for certain pre-existing conditions prior to starting the medication. These conditions include diabetes mellitus, poorly controlled hypertension, osteoporosis, peptic ulcer disease, and cataracts (44). Furthermore, patients already receiving corticosteroids should be closely monitored for the potential development of the aforementioned adverse effects. Assessments include evaluation of bone health via DEXA scans, assessment of the hypothalamic-pituitary-adrenal (HPA) axis through morning cortisol measurement, monitoring of growth using growth curves, evaluation of dyslipidemia and cardiovascular risk through lipid profile testing, and regular ophthalmological examinations to monitor for ophthalmological health. In some cases, the risk of side effects may be reduced in susceptible patients by gradually tapering the corticosteroid dose to the lowest level necessary to achieve therapeutic goals, therefore reducing the likelihood of adverse events (44).

### 4.2 Role of immunomodulators

#### 4.2.1 Methotrexate

Immunomodulators such as methotrexate can be used in the treatment of sarcoidosis as a steroid sparing agent to bypass the steroids side effects. Methotrexate is the most common second line medication used as an alternative to steroids (40) and functions to increase the levels of adenosine, which has anti-inflammatory effects, by inhibiting the conversion of 5-aminoimidazole-4-carboxamide ribonucleotide (AICAR) to formyl AICAR (FAICAR) by inhibiting AICAR transformylase (46). It also functions to inhibit purine and pyrimidine synthesis via inhibiting dihydrofolate reductase, an enzyme that converts dihydrofolate into the active form of folate, tetrahydrofolate. A reduction in purine and pyrimidine results in a reduction in DNA synthesis and replication, leading to a suppression in T cell replication mediating the inflammation in sarcoidosis (47). Methotrexate as a treatment option to sarcoidosis has received some validation showing effectiveness in small case series and a several randomized control trial indicating methotrexate showed effectiveness in the therapy of both acute and chronic sarcoidosis (48–50).

#### 4.2.2 Azathioprine

Azathioprine is another immunomodulator which inhibits purine metabolism similarly to methotrexate albeit via a different pathway and was shown to have equal efficacy to methotrexate in the role of second line treatment to sarcoidosis (48).

Despite their potential, a significant amount of patients experience toxicity from these drugs. In methotrexate, common adverse effects that can arise include leukopenia, fatigue, hepatotoxicity, gastrointestinal distress and infections (38). Azathioprine gives rise to similar side effects; however, infections are more common than in methotrexate users (39).

#### 4.2.3 Leflunomide

Other immunomodulators include leflunomide, a dihydroorotate dehydrogenase inhibitor preventing lymphocyte division similar to the aforementioned drugs (38). Data has shown that the drug displays steroid-sparing effects and significant improvements in pulmonary function (51). One clinical trial of Leflunomide in patients with chronic sarcoidosis found that its efficacy was similar to methotrexate, all while being well tolerated and showing less toxicity. Therefore, it can be inferred that Leflunomide can be used as an alternative to methotrexate in those who cannot tolerate the drug (52).

#### 4.2.4 Mycophenolate mofetil

Mycophenolate mofetil functions via inhibiting purine nucleotide synthesis in lymphocytes and decreases autoantibody production by b-cells (38). A retrospective chart review found no significant changes present in pulmonary function testing pre and post mycophenolate treatment in the retrospective cohort study group. However, a trend in the improvement of DLCO 12 months pre and post drug therapy was identified, and steroid-sparing effects were also noted. Overall, this concluded that mycophenolate mofetil could prove beneficial in those who are intolerant to steroid therapy (53). Additionally, the drug has displayed positive efficacy in the treatment of central nervous system sarcoid in a study investigating the drug’s efficacy against neurosarcoidosis, with the identification of a steroid-sparing effect as well as better tolerability in comparison to other immunosuppressive drugs (54).

### 4.3 Targeted biologic agents

TNF-α is a proinflammatory cytokine which binds to its TNFR1/2, triggering downstream activation of inflammatory signals by activating NF-κB (55). It promotes the recruiting and activation of immune cells. TNF-α contributes to chronic inflammation as a result and leads to the formation and maintenance of granulomas, which is the hallmark of sarcoidosis (56). TNF-α can be inhibited by antagonists such as infliximab, which could reduce granuloma formation and persistence and therefore the severity of disease (55). In a recent retrospective study, infliximab was administered as a second or third line drug for sarcoid with majority of those in the ocular-cardiac-cutaneous-CNS, neurosarcoid, abdominal organ, pulmonary-lymph-nodal and extrapulmonary groups achieving a good response to the drug. However, Infliximab led to a significant number of patients, specifically 36%, falling into serious adverse events being mainly infections, which led to two deaths (57). Additionally, infliximab was found to be effective in refractory sarcoidosis, especially neurologic and cutaneous sarcoidosis (58), while a separate study found greater efficacy in patients with higher TNF-α levels (59). Although the trials in adalimumab are limited, in those done, it has shown good efficacy and relative safety in the treatment of sarcoidosis (60–62). As adalimumab’s mechanism of action is closely related to that of infliximab, it has shown significant efficacy to intolerance to infliximab therapy in patients with sarcoidosis (63). This indicates the importance of further research into these therapies as they show certain promise in the field of sarcoidosis therapeutics.

The decision to transition between or to combine first-line corticosteroids, second-line immunomodulators and/or third-line biologics is multifactorial. Some factors influencing the decision-making process include patient age, comorbidity status, whether or not the side-effect profile is favorable, efficacy of the drug, and the patient’s adherence to the drug. The decision to combine the therapies could arise in cases of aggressive variations of sarcoidosis such as severe neurosarcoidosis and end-stage infiltrative heart disease (38).

### 4.4 Antifibrotics

Antifibrotic drugs such as nintedanib have been shown preclinically to inhibit tyrosine kinases, therefore halting the progression of lung fibrosis (64). One study has shown nintedanib provides significant lowering of FVC for over 100 compared to those in placebo in patients with progressive interstitial lung disease, diarrhea being the commonest side effect. Although results are positive, sarcoid patients were a part of a minority group under the heading of “other fibrosing ILDs” which only comprised of 12% of the study cohort, as a result, it is difficult to draw conclusions as sarcoid disease was not purely tested for (38, 64). Efzofitimod is another antifibrotic drug which functions via binding Neuropilin-2 (NRP2), a receptor which is upregulated in inflammatory insult (65). The binding and inhibition of this receptor was found to inhibit the recruitment of immune cells (66), specifically the recruitment of cells of the myeloid lineage (65). However, the lack of trials in the use of antifibrotic drugs for sarcoid, especially Nintedanib, emphasizes the need for further research in the area in the hopes of recognizing a well-tolerated and efficacious drug.

### 4.5 Future treatment tailored to immunologic findings

Specific mutations underlying the pathophysiology of sarcoidosis can be identified further down the line. The presence of these mutations means more specific drug targets, leading to more efficient therapy with fewer side effects. One example of dysregulation identified in the pathogenesis of sarcoidosis is the mTORC1 pathway (67). This mutation opens the door to drugs directly targeting this area i.e., mTOR inhibitors, with studies already detailing the positive effects of the drug against sarcoid disease (68, 69). Sirolimus, a type of mTOR inhibitor preventing cell cycle progression and limiting immune cell proliferation, has shown reasonable efficacy in cutaneous sarcoidosis, although trials in this area are still needed (70). The lack of trials and testing on these drugs emphasizes the need for further research to develop safer and more targeted downstream drugs for the future treatment of sarcoidosis.

## 5 Discussion

### 5.1 Challenges and future directions

Despite advances in sarcoidosis research, several challenges remain in fully understanding the disease. One significant issue is the etiological heterogeneity of sarcoidosis, where the precise causes and mechanisms remain unclear (71–73). Variations in genetic, environmental, and immunological factors contribute to the diverse manifestations of the disease, making it difficult to develop a universal understanding of its onset and progression (74–77).

Another challenge is the integration of multi-omics data (genomics, transcriptomics, proteomics, and metabolomics) into clinical practice. While these data provide valuable insights into disease mechanisms, translating them into actionable biomarkers or treatment strategies is complex (78, 79). Standardized protocols and comprehensive validation studies are needed to fully unlock the potential of omics data, with the exciting prospect of significantly enhancing clinical decision-making and patient outcomes in the future (80–83).

Looking ahead, the potential for precision medicine in sarcoidosis is promising. There already exists research aiming to detect genetic and molecular differences present in sarcoid disease. For instance, one study detailed certain chromosome linkages to sarcoidosis, i.e., chromosome 5 in African Americans and chromosome 6 in German families, specifically the BTNL2 gene of chromosome 6 being associated with sarcoidosis (84). The identification of pathways involved in the pathogenesis of sarcoidosis, which include the interferon response, T-cell receptor signaling, and the major histocompatibility complex, has also been discussed (85). Similar to how mTOR inhibitors have risen to efficacy against sarcoidosis, the identification of these molecular dysregulations facilitates the future use of drugs inhibiting these pathways, leading to a reduction in granulomatous inflammation and progression of the disease. By leveraging individual genetic profiles, clinical features, and omics data, it may be possible to tailor treatments to the specific needs of patients (82, 83, 86, 87). However, significant hurdles remain, including the need for large-scale longitudinal studies, better diagnostic tools, and an improved understanding of the disease’s pathophysiology to make precision medicine a reality in sarcoidosis care. Furthermore, the lack of a standardized classification system for sarcoidosis subtypes complicates both diagnosis and treatment, adding another layer of complexity to advancing care. The complex interplay between genetic and environmental factors is not yet fully understood (88).

### 5.2 Interdisciplinary collaboration

Given its multisystem nature, interdisciplinary collaboration is essential in sarcoidosis research, as specialists must assess affected organs and optimize treatment. A coordinated, tailored approach is essential to improving diagnosis and management, underscoring the importance of collaborative efforts to advance understanding and care (89, 90).

As part of this effort, understanding the role of environmental and lifestyle factors is critical. The role of tobacco smoking in sarcoidosis remains controversial, and the impact of obesity, physical activity, and diet on sarcoidosis risk, particularly in men, has not been thoroughly studied (91). Additionally, the identification of patients at risk for sarcoidosis-related comorbidities and mortality remains unclear, with treatment decisions typically based on symptom presence or organ involvement rather than predictive factors (91).

## 6 Conclusion

Sarcoidosis remains a complex and heterogeneous disease, presenting significant challenges in both diagnosis and treatment. Its varied clinical manifestations across different organs, coupled with the lack of a definitive diagnostic test, complicate early detection and accurate diagnosis. Moreover, the disease’s unpredictable course necessitates a personalized treatment approach, tailored to the individual patient’s specific symptoms and affected organs. Understanding the underlying etiology of sarcoidosis is crucial for refining diagnostic protocols and developing more effective, targeted therapeutic strategies. Further research into the genetic, environmental, and immunological factors that contribute to the disease could provide valuable insights, potentially leading to more standardized approaches to treatment and improved patient outcomes. The findings of this review underscore the need for continued research into novel biomarkers and therapeutic pathways, which could lead to more precise diagnostic tools and treatment regimens. This will ultimately facilitate earlier detection, more effective management, and improved patient outcomes.
